# Care cascades of diabetes and hypertension among late adolescents in India

**DOI:** 10.7189/jogh.15.04101

**Published:** 2025-03-07

**Authors:** Bijaya Kumar Malik, Amit Kumar Goyal, Suraj Maiti, Sanjay K Mohanty

**Affiliations:** 1 National Population Education Project, DESS, NCERT, New Delhi, India; 2 Department of Population and Development, International Institute for Population Sciences, Mumbai, Maharashtra, India; 3 Department of Economics, Virginia Polytechnic Institute and State University (Virginia Tech), Blacksburg, Virginia, USA

## Abstract

**Background:**

Diabetes and hypertension are the most prevalent morbidities in India and are quickly becoming common among the younger age groups. Adolescents aged 10–19 years, accounting for one-fifth of the country’s population, are at an increasing risk of developing these conditions. We aim to examine the prevalence, awareness, treatment, and control (ATC) of diabetes and hypertension among late adolescents (15–19 years) in India.

**Methods:**

We used microdata of 204 346 late adolescents from India's fifth round of the National Family and Health Survey, 2019–21. We defined hypertensive adolescents as those diagnosed with hypertension or those with a systolic blood pressure (BP) measurement of ≥130 mm Hg, diastolic BP measurements of levels ≥80 mm Hg, or those who used medication to lower BP at the time of the survey. Diabetic adolescents were those diagnosed as such by health professionals, those with glucose levels above 140 mg/dL, or those taking any medication to control high blood glucose levels at the time of the survey. We estimated the age-sex-adjusted prevalence of both conditions and their ATC rates, referred to as cascade care. We used the Erreygers’ Concentration Index to examine the socioeconomic inequality in cascade care. We used multivariable logistic regression to estimate the average marginal effects while controlling for sociodemographic characteristics.

**Results:**

Of 204 346 late adolescents, 27.8% (95% confidence interval (CI) = 27.6, 28.2) had either of the two conditions, with 3.5% (95% CI = 3.4, 3.6) being diabetic and 24.3% (95% CI = 24.0, 24.6) having hypertension. The ATC rate of diabetes was 13.5% (95% CI = 12.4, 14.7), 13.1% (95% CI = 11.9, 14.2), and 12.1% (95% CI = 11.0, 13.3), respectively. For hypertension, the ATC rate was extremely low at 6.2% (95% CI = 5.8, 6.5), 3.5% (95% CI = 3.3, 3.7), and 3.3% (95% CI = 3.1, 3.5), respectively. There was a pro-rich socioeconomic inequality in the prevalence of hypertension and a pro-poor inequality in the prevalence of diabetes among late adolescents. We observed significant variations in both conditions across the regions of India.

**Conclusions:**

The high prevalence and low care cascade levels of diabetes and hypertension among late adolescents in India are concerning. A multipronged strategy that includes screening, diagnosis, and timely interventions at school and home can reduce the burden of hypertension and diabetes among the prospective workforce in India. Sensitising adolescents through school curricula under the New Education Policy (2020) is recommended to reduce the burden of these conditions. We also recommend that longitudinal and intervention studies focussed on this age group be undertaken in the future to help reduce the disease burden.

Hypertension and diabetes are two of the most prevalent non-communicable diseases (NCDs) worldwide, accounting for 218 million and 76 million disability-adjusted life years in 2019, respectively [1]. These conditions are the primary risk factors for many chronic diseases, including cardiovascular and respiratory diseases, and lead to treatment complications [1]. In 2019, an estimated 10.4 million deaths were attributed to hypertension and 1.5 million deaths to diabetes alone. The onset age of both hypertension and diabetes in low- and middle-income countries is much lower than in developed countries [2–4]. Both diseases have been progressing rapidly and have come to affect the working-age population, youth, and adolescents, increasing the risk of premature mortality. Hypertension has its origin in childhood, and children diagnosed with hypertension have a high risk of developing cardiovascular disease and type 2 diabetes in the later stage [5–8].

The late adolescents (15–19 years) have a significantly higher risk of developing hypertension than the early adolescents (10–14 years), possibly because of higher academic pressure, career concerns, and behavioural factors like substance use, unhealthy diet, prolonged exposure to social media, and less physical activities [7,8]. Other leading risk factors of hypertension and diabetes are genetic, metabolic, and lifestyle-related [9,10]. The genetic factors include family history, while the metabolic factors include overweight/obesity and high cholesterol [10]. The behavioural and lifestyle factors are tobacco use, alcohol consumption, substance abuse, physical inactivity, dietary habits, and stress [11].

Most of the risk factors are acquired and due to lifestyle changes during adolescence [12]. Two recent studies showed that nearly half of adolescents in their samples had unhealthy dietary consumption (*i.e.* junk food) and that one-third had inadequate consumption of fruits and vegetables [13,14]. Adolescents are known for experimentation and are vulnerable to adopting certain lifestyles, which may predispose them to NCDs [15]. A study based on the Comprehensive National Nutrition Survey estimated a diabetes prevalence of 0.56% among adolescents in India [16], with the prevalence being higher among boys, those who were obese, those physically not active, and those who consumed unhealthy diets. Quality of life was also found to be lower among adolescents with type 1 diabetes in Bhavnagar, Gujarat [17]. The parents of children who received special diabetes care generally were found to be better educated, while their school was very supportive about their diabetic condition [18]. Several social and economic determinants are associated with type 1 diabetes in childhood [19]. Due to missed diagnoses, low awareness, and limited availability of insulin and blood glucose strips in India and low- and middle-income countries in general, diabetes has been increasing among adolescents. Simultaneously, the inadequacy of medical care and prevention strategies leads to more complications and financial burdens later in life. The growing prevalence of diabetes among adolescents is a matter of concern, and awareness about the disease before its occurrence is the need of the hour [7].

Adolescents aged 10–19 years accounted for over one-fifth of India’s population in 2011 and are growing faster in numbers than the overall population. The official population projection of India suggests that the population of late adolescents (15–19 years) is likely to be 117.6 million by 2026 and 112.7 million by 2036 [20]. While studies on care cascades (defined as awareness, treatment, and control (ATC)) of hypertension and diabetes have been growing in India [21–27], most have focussed on the adult population aged ≥18 years [22,23]. Their findings have suggested lower ATC of both diseases across socioeconomic groups and a robust socioeconomic gradient in the care cascades [21,25]. Only one study focussed on the ≥15 age group for diabetes but did not separately focus on the late adolescents, that is, those in the 15–19-year age group [21].

In this study, we address this critical gap by examining the care cascades of diabetes and hypertension among late adolescents (15–19 years) in India. We conceptualised it with the following rationale: first, diabetes and hypertension are two of the most prevalent diseases in India and are increasingly becoming common among adolescents [28]. Second, adolescents are in a transitional stage and undergo several changes in their lives concerning education, employment, and physiological changes. If diabetes and hypertension remain unchecked, they will carry these conditions for a longer period and contribute a larger share to the disease burden of NCDs in India. Third, both diseases can be prevented and managed by creating awareness and making treatment accessible.

We aimed to estimate the prevalence and ATC of diabetes and hypertension among late adolescents in India and to examine the socioeconomic and regional inequalities in disease prevalence and care cascades.

## METHODS

### Data source

We used the cross-sectional data from the fifth round of the National Family Health Survey (NFHS-5), conducted in two phases from June 2019 to April 2021 [29]. The NFHS-5 used a stratified two-stage sampling design. For rural areas, villages were selected as primary sampling units (PSUs), while census enumeration blocks were selected for this role for urban areas. Specifically, PSUs were selected using probability proportional to size sampling. Then, a complete household mapping and listing was conducted in each selected PSU, from which 22 households were randomly selected using systematic sampling. Written informed consent was obtained from all the respondents, and ethical clearance was obtained from the institutional review boards of the International Institute for Population Studies and ICF International [29].

### Inclusion/exclusion criteria

**Figure 1:**
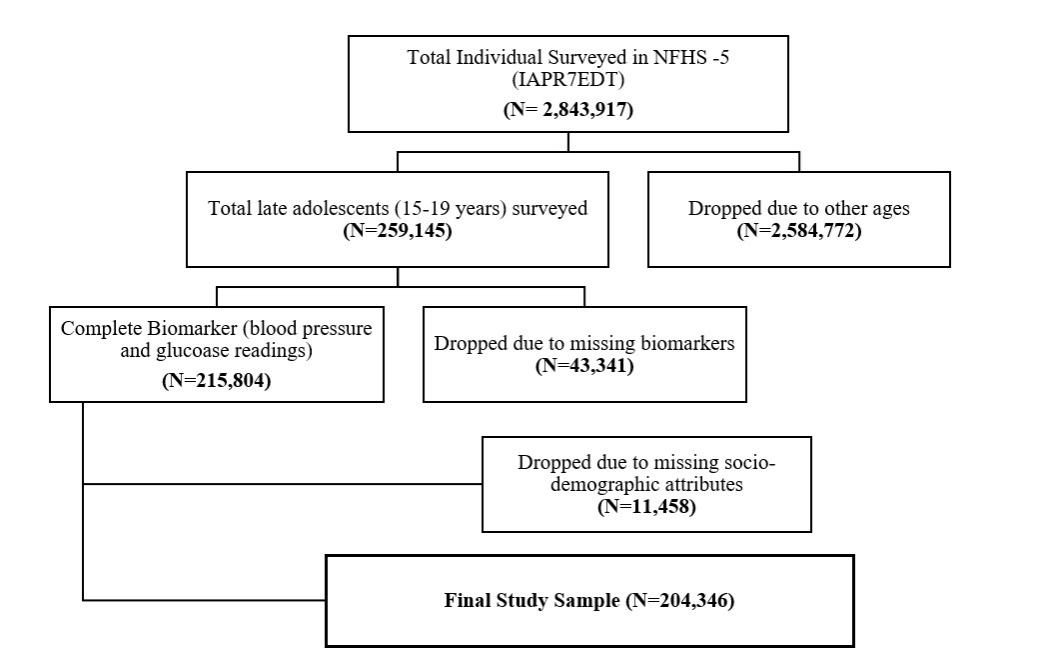
Flowchart of sample selection.

We included a sub-sample of late adolescents aged 15–19 years among the 636 699 surveyed households and used the household member recode data (IAPR7EDT). Out of 2 843 917 surveyed individuals, we included 259 145 who met the age criteria. We excluded individuals outside this age range and those with missing data on key variables (*i.e.* blood pressure (BP), blood glucose, or sociodemographic characteristics) (**Figure 1**). Thus, our complete case analytic sample consisted of 204 346 individuals.

### Outcome variables

#### Hypertension

In the NFHS-5, BP was measured using an Omron BP monitor (Omron Healthcare, Illinois, USA) [29]. Each participant had three BP readings taken, with a five-minute interval between measurements. We considered the average of the three BP readings to determine if a participant had hypertension. When a measurement was missing, we relied on the average of the remaining two measurements. An adolescent was defined as hypertensive if they met any of the following criteria:

− diagnosed with hypertension by a doctor or any other health professional;
− measured systolic BP level ≥130 mmHg or diastolic BP level ≥80 mmHg at the time of the survey;
− current use of medication to lower BP.

These thresholds were based on the guidelines given by the American Academy of Pediatrics (2017) and the Indian Academy of Pediatrics, are validated in the Indian context, and are lower than the adult (≥18 years) BP threshold (Table S1 in the **Online Supplementary Document**) [30–32]

Among the hypertensive respondents, ‘aware’ was defined as one who responded with ‘yes’ to the question *‘*Do you currently have hypertension?*’*, ‘treated’ was defined as one who responded with ‘yes’ to the question ‘Have you sought treatment for hypertension?’, and ‘control’ was defined as one was being ‘treated’ for hypertension and had recorded systolic BP<130 mm Hg and diastolic BP<80 mm Hg at the time of the survey [26].

#### Diabetes

Random blood glucose testing was conducted by trained health investigators using the Accu-Chek Performa glucometer (Roche Diabetes Care, Inc.) with glucose test strips on all adults aged ≥15 years. An adolescent was classified as diabetic if they met any of the following criteria:

− told by a doctor or any other health professional that they have high blood glucose;
− measured glucose levels >140 mg/dL at the time of the survey;
− taking any medication to control high blood glucose levels.

Among the diabetic respondents, ‘aware’ was defined as one who responded with ‘yes’ to the question *‘*Do you currently have diabetes?*’*, ‘treated’ was defined as one who responded with ‘yes’ to the question ‘Have you sought diabetes treatment’, and ‘control’ was defined as one who was being ‘treated’ for diabetes and had a recorded glucose level <140 mg/dL at the time of the survey [21,29]. The cut-offs for blood glucose levels used in the study to identify adolescents with diabetes were as per the NFHS-5 report [29].

#### Covariates

We used several potential socioeconomic and demographic confounders in this study. First among them was the wealth index, a composite variable based on housing, household amenities, and consumer durables to measure the economic status of households [33]. The wealth score was computed from household assets, housing, and household features by using the principal component analysis separately for rural and urban areas and then combined [33]. Demographic characteristics included age (15–19 years), sex (male/female), place of residence (rural/urban), and educational level (no education, primary, secondary, and higher secondary and above). We also included household characteristics such as size (<3, 4–6, and ≥7 members), caste (scheduled tribe, scheduled caste, other backward classes, and others), religious affiliations (Hindu, Muslim, Christian, and others), marital status (currently married/not currently married), and health insurance (yes/no). We additionally considered lifestyle characteristics like the use of alcohol (yes/no) and tobacco (yes/no).

#### Statistical analysis

We estimated the age-sex-adjusted prevalence of hypertension and diabetes along with their cascades of care, stratified by sociodemographic characteristics and across the regions of India. Age and sex adjustment was performed to standardise the prevalence estimates, enabling robust comparisons between groups while controlling for the effects of age and sex composition [21,22]. To quantify inequality in the outcome variables by economic status, we calculated the age-sex standardised Erreygers Concentration Index (ECI) [34]. The ECI is defined as *ECI* = 8*cov*(*y_i_*, *R_i_*), where y_i_ is the health variable for individual i (bounded between 0 and 1), and R_i_ is the fractional rank of individual i in the socioeconomic distribution. The ECI ranges from −1 to +1, with 0 indicating no inequality. It measures the degree of socioeconomic inequality across the full distribution of wealth index, providing an adjusted and standardised distribution of the prevalence of the (ill) health condition. A positive (negative) concentration index indicates a higher prevalence among richer (poorer) individuals, termed pro-rich (pro-poor) inequality.

We employed multivariable logistic regression models to estimate the adjusted marginal effects of covariates on the respective outcome variables. The general form of the model is *logit*(*p*) = *β*_0_ + *β*_1_*X*_1_ + *β*_2_*X*_2_ + … + *β*_k_*X*_ₖ_, where *p* is the probability of the outcome, *X*_1_ to *X_k_* are predictor variables, and *β*_0_ to *β*_k_ are regression coefficients. All estimates, including prevalence, ECI values, and logit regression marginal effects, are presented with 95% confidence intervals (CIs). We used Stata, version 17.1 (StataCorp, College Station, Texas, US) and *R*, 4.4.2 (R Core Team, Vienna, Austria) to perform the statistical analysis and visualise the maps.

## RESULTS

In our complete case analytic sample (**Figure 1**), most of the participants were female (53.2%), lived in households with 4–6 members (72.6%), and resided in rural areas (71.6%). Most (84.2%) had secondary education, and 83% practised Hinduism (Table S2 in the **Online Supplementary Document**). Few participants used tobacco (3.6%) and alcohol (1.1%). The prevalence of diabetes among the late adolescents was 3.5% (95% CI = 3.4–3.6). The ATC rates among adolescents with diabetes were 13.5% (95% CI = 12.4–14.7), 13.1% (95% CI = 11.9–14.2), and 12.1% (95% CI = 11.0–13.3) respectively. Although awareness increased with age, treatment and control were higher at younger ages among the late adolescents. Diabetes was more prevalent among males (3.6%; 95% CI = 3.4–3.8) than females (3.4%; 95% CI = 3.3–3.6). It had a strong economic gradient, with the highest prevalence among the poorest quintile at 3.7% (95% CI = 3.4–3.9) (**Table 1**), while its prevalence increased with age, household size, and level of education (Figure S5 and Table S3 in the **Online Supplementary Document**). The mean blood glucose level was 158.5 (standard deviation (SD) = 0.6) among the late adolescents and varied with age (Figure S4 in the **Online Supplementary Document**).

**Table 1:** Age-sex adjusted prevalence, awareness, treatment and control of diabetes among late adolescents in India, 2019–21

	Prevalence*				Awareness†				Treatment†				Control†				
	**% (95% CI)**		***F*-statistics (*P*-value)**		**% (95% CI)**		***F*-statistics (*P*-value)**		**% (95% CI)**		***F*-statistics (*P*-value)**		**% (95% CI)**		***F*-statistics (*P*-value)**	
**Overall**		3.5 (3.4, 3.6)				13.5 (12.4, 14.7)				13.1 (11.9, 14.2)				12.1 (11.0, 13.3)			
**Age**				13.17 (0.00)				0.73 (0.57)				1.57 (0.18)				1.75 (0.14)	
15		3.0 (2.7, 3.2)				13.2 (10.6, 15.8)				14.1 (11.6, 16.6)				13.7 (11.2, 16.2)			
16		3.3 (3.1, 3.5)				14.9 (12.4, 17.4)				14.5 (12.1, 16.9)				13.0 (10.7, 15.3)			
17		3.4 (3.2, 3.7)				12.7 (10.4, 14.9)				14.4 (11.8, 17.0)				13.6 (11.0, 16.2)			
18		3.6 (3.4, 3.9)				14.1 (11.9, 16.4)				11.7 (9.7, 13.7)				10.8 (8.9, 12.8)			
19		4.3 (4.0, 4.6)				12.6 (10.4, 14.8)				11.3 (8.5, 14.1)				10.2 (7.4, 13.0)			
**Household size**				3.05 (0.05)				0.86 (0.42)				0.26 (0.77)				0.11 (0.90)	
<3		3.7 (3.4, 4.1)				15.6 (12.2, 18.9)				13.3 (10.2, 16.4)				12.3 (9.2, 15.3)			
4–6		3.4 (3.3, 3.6)				13.2 (11.9, 14.6)				12.8 (11.5, 14.0)				12.0 (10.7, 13.2)			
≥7		3.8 (3.5, 4.1)				13.1 (10.5, 15.8)				14.1 (10.5, 17.7)				12.8 (9.3, 16.3)			
**Sex**				2.52 (0.11)				0.15 (0.70)				1.48 (0.22)				1.71 (0.19)	
Female		3.4 (3.3, 3.6)				13.7 (12.2, 15.2)				12.4 (10.7, 14.0)				11.4 (9.8, 13.0)			
Male		3.6 (3.4, 3.8)				13.3 (11.7, 14.9)				13.8 (12.2, 15.3)				12.9 (11.4, 14.4)			
**Residence**				2.29 (0.13)				1.27 (0.26)				0.06 (0.81)				0.13 (0.72)	
Urban		3.4 (3.1, 3.6)				14.7 (12.2, 17.3)				13.3 (10.5, 16.1)				12.5 (9.8, 15.3)			
Rural		3.6 (3.4, 3.7)				13.1 (11.8, 14.4)				13.0 (11.8, 14.1)				12.0 (10.8, 13.1)			
**Education level**				0.65 (0.59)				3.91 (0.01)				2.61 (0.05)				1.92 (0.12)	
No education		3.4 (2.8, 4.0)				7.7 (4.1, 11.4)				8.5 (4.8, 12.1)				8.2 (4.5, 11.8)			
Primary		3.2 (2.8, 3.7)				15.6 (11.1, 20.0)				11.5 (7.9, 15.2)				11.2 (7.6, 14.8)			
Secondary		3.5 (3.4, 3.7)				13.4 (12.2, 14.7)				13.6 (12.2, 14.9)				12.5 (11.2, 13.9)			
Higher secondary and above		3.5 (3.0, 3.9)				15.9 (11.3, 20.4)				10.4 (6.7, 14.2)				9.9 (6.2, 13.5)			
**Caste**				1.57 (0.19)				2.54 (0.05)				0.17 (0.92)				0.23 (0.87)	
Schedule caste		3.4 (3.2, 3.7)				14.3 (11.9, 16.7)				12.4 (10.2, 14.6)				11.6 (9.5, 13.7)			
Schedule tribe		3.3 (3.0, 3.6)				12.3 (9.2, 15.4)				13.1 (10.3, 15.7)				12.6 (10.0, 15.3)			
OBC		3.5 (3.4, 3.7)				14.7 (12.9, 16.4)				13.1 (11.5, 14.7)				12.0 (10.4, 13.5)			
Others		3.7 (3.4, 4.0)				10.8 (8.5, 13.2)				13.7 (10.7, 16.8)				12.9 (9.9, 15.9)			
**Religion**				2.09 (0.10)				1.39 (0.24)				0.98 (0.40)				0.36 (0.78)	
Hindu		3.5 (3.4, 3.6)				13.7 (12.4, 15.0)				12.9 (11.8, 14.1)				12.2 (11.0, 13.3)			
Muslim		3.8 (3.4, 4.1)				11.4 (8.2, 14.5)				12.4 (8.2, 16.6)				11.1 (7.0, 15.2)			
Christan		3.1 (2.5, 3.8)				20.7 (11.4, 30.0)				17.5 (9.2, 25.8)				14.6 (7.1, 22.1)			
Others		3.0 (2.5, 3.5)				13.7 (7.7, 19.8)				17.7 (10.8, 24.6)				14.8 (8.0, 20.6)			
**Currently married**				3.71 (0.05)				1.3 (0.25)				6.23 (0.01)				4.41 (0.04)	
No		3.5 (3.3, 3.6)				13.3 (12.1, 14.5)				13.5 (12.2, 14.8)				12.5 (11.2, 13.7)			
Yes		4.0 (3.5, 4.4)				15.9 (11.6, 20.2)				8.8 (5.7, 12.0)				8.6 (5.4, 11.8)			
**Wealth quintile**				3.81 (<0.001)				4.21 (0.00)				0.54 (0.71)				0.78 (0.54)	
Poorest		3.7 (3.4, 3.9)				10.1 (8.2, 12.0)				12.8 (10.6, 15.0)				11.9 (9.8, 14.0)			
Poorer		3.8 (3.5, 4.0)				12.8 (10.6, 15.0)				13.2 (10.5, 15.9)				12.9 (10.2, 15.6)			
Middle		3.5 (3.3, 3.7)				15.0 (12.4, 17.5)				12.3 (10.0, 14.6)				11.0 (8.8, 13.2)			
Richer		3.3 (3.1, 3.6)				15.6 (12.9, 18.3)				12.5 (10.1, 14.8)				11.2 (9.0, 13.4)			
Richest		3.1 (2.8, 3.4)				15.4 (12.2, 18.7)				15.2 (11.8, 18.6)				14.1 (10.7, 17.4)			
**Tobacco use**				1.16 (0.28)				0.37 (0.54)				2.12 (0.15)				1.7 (0.19)	
No		3.5 (3.4, 3.6)				13.5 (12.3, 14.7)				12.9 (11.7, 14.1)				12.0 (10.9, 13.1)			
Yes		3.2 (2.7, 3.7)				15.2 (9.6, 20.9)				17.4 (11.4, 23.4)				15.9 (10.1, 21.7)			
**Alcohol use**				2.55 (0.11)				0.15 (0.70)				1.13 (0.29)				0.72 (0.40)	
No		3.5 (3.4, 3.6)				13.5 (12.4, 14.7)				13.1 (12.0, 14.3)				12.2 (11.0, 13.3)			
Yes		2.8 (1.8, 3.7)				11.7 (2.5, 21.0)				8.4 (0.1, 17.0)				8.4 (0.1, 17.0)			
**Health insurance**				0.00 (0.99)				2.15 (0.14)				0.06 (0.81)				0.12 (0.73)	
No		3.5 (3.4, 3.7)				12.8 (11.3, 14.3)				13.2 (11.6, 14.8)				12.3 (10.7, 13.9)			
Yes		3.5 (3.3, 3.7)				14.5 (12.7, 16.3)				12.9 (11.2, 14.6)				11.9 (10.3, 13.5)			

CI – confidence interval, OBC – other backward class

*The number of participants was 204 346.

†The number of participants was 6625.

The prevalence of hypertension was 24.3% (95% CI = 24.0, 24.6) in the overall sample (**Table 2**). The prevalence rate of awareness was 6.2% (95% CI = 5.8, 6.5), that of treatment was 3.5% (95% CI = 3.3, 3.7), while that of control was 3.3% (95% CI = 3.1, 3.5). Hypertension increased with age, with the highest prevalence rate occurring at 19 years (29.2%; 95% CI = 28.5, 29.8). Awareness increased with age, whereas treatment and control decreased with age. Hypertension was more prevalent among males (27.6%; 95% CI = 27.2, 28.0) compared to females (21.4%; 95% CI = 21.0, 21.7). However, females were more aware and had better control and treatment of hypertension than late adolescent males. Late adolescents belonging to the richest quintile (25.9%; 95% CI = 25.1, 26.7) had a higher prevalence than those belonging to the poorest quintile (24.1%; 95% CI = 23.6, 24.6). Prevalence was higher among late adolescents who used tobacco (24.4%; 95% CI = 23.2, 25.6), who consumed alcohol (26.2%; 95% CI = 24.1, 28.4%), and who had no health insurance (24.9%; 95% CI = 24.5, 25.3%). The average systolic BP was 136.7 (SD = 0.1), while the average diastolic BP was 82.0 (SD = 0.0) and also varied with age (Figure S4 and Table S3 in the **Online Supplementary Document**)

**Table 2:** Age-sex adjusted prevalence, awareness, treatment and control of hypertension among late adolescents in India, 2019–21

	Prevalence*				Awareness†				Treatment†				Control†				
	**% (95% CI)**		***F*-statistics (*P*-value)**		**% (95% CI)**		***F*-statistics (*P*-value)**		**% (95% CI)**		***F*-statistics (*P*-value)**		**% (95% CI)**		***F*-statistics (*P*-value)**	
**Overall**		24.3 (24.0, 24.6)				6.2 (5.8, 6.5)				3.5 (3.3, 3.7)				3.3 (3.1, 3.5)			
**Age**				175.75 (<0.001)				4.94 (<0.001)				2.51 (0.040)				2.58 (0.040)	
15		19.8 (19.3, 20.4)				5.6 (4.9, 6.3)				4.1 (3.5, 4.7)				3.9 (3.3, 4.5)			
16		21.8 (21.3, 22.4)				5.5 (4.9, 6.2)				3.6 (3.1, 4.1)				3.5 (3.0, 4.0)			
17		23.8 (23.2, 24.4)				6.0 (5.3, 6.7)				3.6 (3.1, 4.1)				3.3 (2.9, 3.8)			
18		27.0 (26.5, 27.6)				6.2 (5.6, 6.8)				3.1 (2.7, 3.5)				2.9 (2.6, 3.3)			
19		29.2 (28.5, 29.8)				7.4 (6.7, 8.1)				3.2 (2.7, 3.7)				3.0 (2.6, 3.5)			
**Household size**				7.57 (<0.001)				1.62 (0.200)				1.64 (0.190)				2.78 (0.060)	
<3		24.2 (23.5, 25.0)				6.2 (5.4, 7.1)				3.4 (2.7, 4.1)				3.3 (2.6, 4.0)			
4–6		24.0 (23.7, 24.4)				6.0 (5.6, 6.4)				3.6 (3.3, 3.8)				3.4 (3.1, 3.7)			
≥7		25.5 (24.8, 26.2)				6.8 (6.0, 7.6)				3.1 (2.6, 3.6)				2.8 (2.3, 3.2)			
**Sex**				608.3 (<0.001)				211.68 (<0.001)				66.91 (<0.001)				79.25 (<0.001)	
Female		21.4 (21.0, 21.7)				8.5 (8.0, 9.1)				4.5 (4.1, 4.8)				4.3 (4.0, 4.7)			
Male		27.6 (27.2, 28.0)				4.1 (3.8, 4.5)				2.6 (2.3, 2.9)				2.4 (2.1, 2.6)			
**Residence**				4.94 (0.030)				1.38 (0.240)				5.35 (0.020)				3.08 (0.080)	
Urban		24.9 (24.2, 25.6)				5.8 (5.1, 6.5)				3.0 (2.6, 3.5)				3.0 (2.5, 3.4)			
Rural		24.0 (23.7, 24.4)				6.3 (5.9, 6.7)				3.7 (3.4, 3.9)				3.4 (3.2, 3.7)			
**Education level**				6.25 (<0.001)				0.59 (0.620)				0.82 (0.480)				0.99 (0.400)	
No education		26.3 (24.9, 27.6)				5.5 (4.3, 6.8)				3.1 (2.2, 4.1)				3.1 (2.2, 4.0)			
Primary		26.0 (24.8, 27.1)				6.4 (5.2, 7.7)				3.4 (2.6, 4.3)				3.3 (2.4, 4.1)			
Secondary		24.1 (23.8, 24.5)				6.2 (5.8, 6.6)				3.5 (3.3, 3.8)				3.4 (3.1, 3.6)			
Higher secondary and above		23.7 (22.7, 24.7)				5.8 (4.8, 6.8)				2.9 (2.1, 3.8)				2.7 (1.9, 3.4)			
**Caste**				7.37 (<0.001)				14.2 (<0.001)				1.43 (0.230)				1.28 (0.280)	
Schedule caste		24.1 (23.5, 24.7)				6.4 (5.7, 7.0)				3.2 (2.8, 3.6)				3.1 (2.6, 3.5)			
Schedule tribe		25.7 (24.9, 26.5)				4.3 (3.6, 5.0)				3.2 (2.7, 3.8)				3.0 (2.5, 3.6)			
OBC		23.8 (23.4, 24.2)				7.0 (6.4, 7.5)				3.7 (3.4, 4.1)				3.5 (3.2, 3.9)			
Others		24.9 (24.3, 25.6)				5.3 (4.6, 5.9)				3.4 (2.9, 3.9)				3.2 (2.7, 3.7)			
**Religion**				17.53 (<0.001)				0.64 (0.590)				1.6 (0.190)				1.68 (0.170)	
Hindu		24.1 (23.8, 24.4)				6.3 (5.9, 6.6)				3.5 (3.2, 3.7)				3.3 (3.0, 3.5)			
Muslim		25.3 (24.4, 26.2)				5.8 (4.9, 6.7)				3.4 (2.8, 4.0)				3.3 (2.7, 3.9)			
Christan		21.4 (19.9, 23.0)				5.3 (3.6, 7.0)				5.3 (3.5, 7.1)				5.1 (3.4, 6.9)			
Others		28.8 (27.3, 30.4)				6.1 (4.8, 7.4)				2.9 (1.7, 4.1)				2.8 (1.6, 3.9)			
**Currently married**				0.73 (0.39)				78.66 (<0.001)				10.94 (<0.001)				10.33 (<0.001)	
No		24.3 (24.0, 24.6)				5.5 (5.2, 5.9)				3.3 (3.1, 3.6)				3.2 (2.9, 3.4)			
Yes		23.9 (22.9, 24.9)				12.5 (10.9, 14.0)				5.1 (4.1, 6.1)				4.8 (3.8, 5.8)			
**Wealth quintile**				6.22 (<0.001)				4.01 (<0.001)				2.86 (0.020)				2.94 (0.020)	
Poorest		24.1 (23.6, 24.6)				6.2 (5.5, 6.8)				4.0 (3.5, 4.5)				3.8 (3.4, 4.3)			
Poorer		23.7 (23.1, 24.2)				6.9 (6.2, 7.6)				3.8 (3.3, 4.2)				3.6 (3.1, 4.0)			
Middle		23.8 (23.2, 24.4)				6.5 (5.8, 7.1)				3.3 (2.9, 3.8)				3.1 (2.7, 3.6)			
Richer		24.4 (23.8, 25.1)				5.1 (4.5, 5.8)				3.1 (2.6, 3.6)				2.9 (2.4, 3.4)			
Richest		25.9 (25.1, 26.7)				6.0 (5.1, 6.8)				3.1 (2.5, 3.6)				2.9 (2.3, 3.4)			
**Tobacco use**				0.01 (0.910)				0.11 (0.740)				2.24 (0.130)				2.04 (0.150)	
No		24.3 (24.0, 24.6)				6.2 (5.8, 6.5)				3.5 (3.3, 3.7)				3.3 (3.1, 3.5)			
Yes		24.4 (23.2, 25.6)				6.4 (4.9, 8.0)				2.7 (1.7, 3.7)				2.6 (1.6, 3.6)			
**Alcohol use**				3.16 (0.080)				0.02 (0.880)				10.4 (<0.001)				11.72 (<0.001)	
No		24.3 (24.0, 24.6)				6.2 (5.8, 6.5)				3.5 (3.3, 3.7)				3.3 (3.1, 3.5)			
Yes		26.2 (24.1, 28.4)				6.4 (3.3, 9.5)				1.8 (0.8, 2.8)				1.7 (0.7, 2.6)			
**Health insurance**				27.47 (<0.001)				6.37 (0.010)				0.92 (0.340)				1.2 (0.270)	
No		24.9 (24.5, 25.3)				6.5 (6.0, 7.0)				0.2 (3.9, 3.7)				3.4 (3.1, 3.7)			
Yes		23.5 (23.0, 23.9)				5.7 (5.2, 6.2)				0.2 (3.7, 2.8)				3.2 (2.8, 3.5)			

CI – confidence interval, OBC – other backward class

*204 346 participants.

†52 094 participants.

Compared to stage 1 hypertension, the prevalence of stage 2 hypertension was lower among the late adolescents; however, ATC was higher among the stage 2 hypertensives (Tables S1 and S5 in the **Online Supplementary Document**) The ATC of diabetes and hypertension among the late adolescents was lower than that of the adult (≥20 years) population (Figures S5 and S6 in the **Online Supplementary Document**)

The prevalence of the co-occurrence of hypertension and diabetes was 1.1% (95% CI = 1.0, 1.1), with this comorbidity increasing with age and was higher among males and urban residents (Table S4 in the **Online Supplementary Document**). The prevalence of having either hypertension or diabetes was 27.9% (95% CI = 27.6, 28.2) among all late adolescents.

Hypertension alone, hypertension or diabetes, and the comorbidity of hypertension and diabetes showed a pro-rich inequality, whereas diabetes showed a pro-poor inequality (**Table 3**; Figure S1 in the **Online Supplementary Document**). In contrast, ATC of hypertension exhibited pro-poor inequality, whereas ATC of diabetes exhibited a pro-rich inequality among late adolescents (Figures S2 and S3 in the **Online Supplementary Document**).

**Table 3:** ECI of chronic conditions among late adolescents

	n	ECI index (95% CI)	*P*-value
**Hypertension**	204 346	0.005933 (0.0027, 0.0091)	<0.001
Awareness	52 094	–0.003414 (–0.0069, 0.0001)	0.0575
Treatment	52 094	–0.004250 (–0.0068, –0.0017)	0.0013
Control	52 094	–0.004176 (–0.0067, –0.0017)	0.0011
**Diabetes**	204 346	–0.002368 (–0.0037, –0.0011)	<0.001
Awareness	6625	0.022265 (0.0106, 0.0340)	<0.001
Treatment	6625	0.003993 (–0.0090, 0.0170)	0.5483
Control	6625	0.001824 (–0.0109, 0.0145)	0.7783
**Hypertension and diabetes**	204 346	0.000506 (–0.0002, 0.0012)	0.1676
**Hypertension or diabetes**	204 346	0.0025 (–0.0005, 0.0055)	0.104

CI – confidence interval, ECI – Erreygers’ concentration index

**Figure 2:**
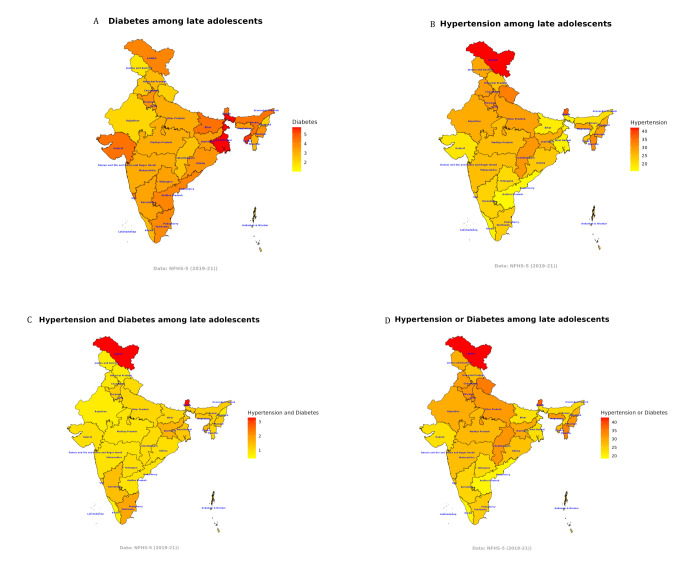
Spatial distribution of hypertension/diabetes prevalence in late adolescents in India, 2019–21. **Panel A.** Diabetes. **Panel B.** Hypertension. **Panel C.** Hypertension and diabetes. **Panel D.** Hypertension or diabetes.

The prevalence of diabetes varied significantly, with West Bengal (5.9%) having the highest prevalence, followed by Tripura (5.2%), and Sikkim (4.7%) (**Figure 2**, Panel A; Table S7 in the **Online Supplementary Document**). Meanwhile, Chandigarh (1.0%), Jammu and Kashmir (1.8%), and Delhi (1.8%) had the lowest prevalence of diabetes. The prevalence of hypertension was highest among the late adolescents of Ladakh (41.6%), followed by Sikkim (36.8%), and Arunachal Pradesh (36.1%), and was lowest among the late adolescents of Lakshadweep (16%), Andhra Pradesh (16.6%), and Kerala (17.6%) (**Figure 2**, Panel B; Table S7 in the **Online Supplementary Document**).

**Figure 3:**
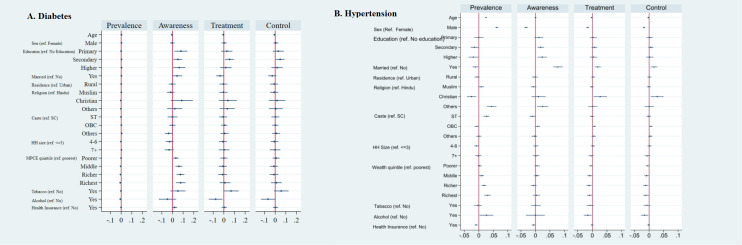
Average marginal effects on probability of hypertension/diabetes and on probabilities of ATC among those with hypertension/diabetes among late adolescents in India, 2019–21. **Panel A.** Diabetes. **Panel B.** Hypertension.

The prevalence of diabetes among late adolescents increased by 0.3 percentage points (pp) for each unit increase in age and was higher by 0.3 pp among males compared to females (**Figure 3**, Panel A; Table S8 in the **Online Supplementary Document**). The richest fifth of late adolescents had a 0.8 pp lower prevalence of diabetes but a 7.8 pp higher awareness than the poorest fifth, showing socioeconomic inequality. Late adolescents who consumed alcohol had 7.8 pp and 6.8 pp lower treatment and control of diabetes, respectively.

The prevalence of hypertension increased by 2.5 pp for each unit increase in age (**Figure 3**, Panel B; Table S9 in the **Online Supplementary Document**). Hypertension treatment and control, however, decreased by 0.3 pp. The prevalence was higher among males by 6.2 pp, but males had lower awareness (3.3 pp), treatment (1.6 pp), and control (1.7 pp) compared to females. Compared to diabetes prevalence among the late adolescents, hypertension prevalence was 3.0 pp higher among the richest fifth compared to the poorest fifth, signifying the pro-rich inequality. Also, hypertension prevalence was lower among late adolescents who had health insurance (0.9 pp), were Christians (2.5 pp), were married (1.1 pp), had secondary (1.4 pp) and higher secondary and above (1.9 pp) educational attainment. The prevalence was higher among late adolescents who belonged to the schedule tribe category (2.7 pp) and those who consumed alcohol (2.6 pp) than their respective counterparts.

## DISCUSSION

The health and well-being of adolescents are paramount for the development of any nation, as they represent tomorrow's workforce, future parents, and key nation-building stakeholders. Adolescence is associated with physiological changes, cognitive development, educational attainment, career challenges, familial responsibility, and first entry into the labour market. While adolescent health in India has received programmatic and research attention from time to time, adolescents’ health needs have changed over time, as this group continued to evolve. The increasing prevalence of hypertension and diabetes among adolescents is an emerging health risk that warrants urgent attention. The National Education Policy (NEP) 2020 explicitly targets these age groups to control these diseases in the educational framework [35].

Our study revealed several important findings regarding hypertension and diabetes among late adolescents (15–19 years) in India. First, we found that the age and sex-adjusted prevalence of diabetes was 3.5% (95% CI = 3.4, 3.6), while that of hypertension was 24.3% (95% CI = 24.0, 24.6). Second, among the late adolescents who had diabetes, only 13.0% each were either aware of, treated for, or in control of this condition. The figures were much lower in the case of hypertensives with 6.2% (95% CI = 5.8, 6.5) awareness and only 3.0% each receiving treatment and being in control. Third, there was a pro-rich inequality in the prevalence of hypertension, but a pro-poor inequality in the prevalence of diabetes among late adolescents. The inequality gradient among co-morbid hypertensive and/or diabetic late adolescents was pro-rich. Fourth, the ATC of hypertension and diabetes among adolescents decreased with age, though prevalence showed an increasing pattern. ATC was higher among girls, though the prevalence was higher among late adolescent boys. Fifth, we observed significant state-wise and region-specific variations in diabetes and hypertension prevalence among late adolescents, whereby the prevalence of both diseases was much higher in the southern states of India compared to the northern states of India.

Our estimates of diabetes prevalence are consistent with those of previous studies [21,30]. However, our estimates of hypertension are higher than the pooled prevalence found in a recent study [28,36]. One plausible reason is the difference in the range of cutoffs of BP of the adolescents. Our cutoff was robust as the Academy of Pediatrics and the Indian Academy of Pediatrics recommend it [31,32]. However, the socioeconomic patterning of diabetes differed from that of the adult population. In the case of the adult population, the prevalence of diabetes was higher among the rich, while in the case of late adolescents, it was higher among the poorer subgroup [21]. The ATC estimates for the late adolescents were much lower than for the adult population (≥20). Nevertheless, the socioeconomic inequality in ATC was similar for late adolescents and the overall population [21–23].

We found few studies across the globe with a focus on the care cascades of diabetes and hypertension among late adolescents. However, studies focussing on adults (not specifically late adolescents) provide some context [6,21,27,37]. For instance, a systematic review reported global diabetes awareness to be at 60%, treatment at 45%, and control at 22% among adults, with higher prevalence in high-income countries [38]. In contrast, our findings among Indian adolescents showed lower ATC. Regarding hypertension, a study across seven sub-Saharan African countries found an average adult prevalence of 25%, with the prevalence of awareness standing at 40%, of treatment at 50% among those aware, and of control at 50% among those treated [39]. These figures are higher than those observed among late adolescents in India, suggesting that the health status of Indian adolescents is comparatively better than in other low- and middle-income countries, but poorer than the global average.

Given the significant regional variations in diabetes and hypertension prevalence among late adolescents, a ‘one-size-fits-all’ approach is unlikely to be effective. Health interventions should be tailored to the specific needs of each state, considering local dietary habits and lifestyle factors. For instance, interventions in West Bengal, Tripura, and Sikkim might focus on promoting healthy diets and increasing physical activity to combat diabetes, while those in Ladakh, Sikkim, and Arunachal Pradesh could focus on hypertension management, potentially including salt reduction campaigns and ensuring access to affordable medication. Indian states also vary significantly in terms of health infrastructure, which makes the screening and management of diabetes and hypertension difficult. Healthcare interventions should also consider the socioeconomic context and address potential barriers to care faced by adolescents from lower socioeconomic backgrounds (*e.g.* Bihar, West Bengal, and Chhattisgarh), as they may be disproportionately vulnerable to higher severity [23,37].

We place our findings in the context of the National Curriculum Framework for School Education (NCF-SE) 2023, the NEP 2020, and the National Health Policy 2017. The NCF-SE and the NEP are the leading government policies that advocate for and analyse school education policies and programmes. The NEP 2020 emphasises that it is important to ensure that adolescents are not ensnared by NCDs and are made aware of the detrimental effects of NCDs on their future health. The NCF-SE, established in 2023, is instrumental in preventing adolescent NCDs. Although NCF-SE primarily focusses on higher education and research, it can be implemented across all levels of schooling [35,40]. The National Health Policy 2017 also emphasises health education and promotion as part of the curriculum in schools [41].

Increasing the awareness and knowledge among adolescents about hypertension and diabetes and their risk factors is an essential part of the population-based prevention strategy. Assessing and appropriately disseminating knowledge of the modifiable risk factors early is a crucial preventive educational approach [42]. Early diagnosis and timely intervention can be effective steps in controlling and preventing disease complications and reducing the burden of hypertension and diabetes. Regular BP screening is mandatory to check for disease complications at an early stage. This calls for a special screening programme for adolescents per the cut-offs recommended by the Indian Academy of Pediatrics [31]. We suggest that the screening programme be conducted at the school/college level and be made part of the school/college curriculum. Improving country-level surveillance and monitoring systems should be a top priority to cope with the expanding need for policies, legislation, services, and infrastructure to prevent and control NCDs [43].

Intervention programmes must encourage students to adopt healthy lifestyle practices such as improving dietary intake, increasing physical activity, avoiding a sedentary lifestyle, and avoiding stress. Private school students from higher-income families, who can afford a healthy lifestyle, are also adopting unhealthy behaviour, which is increasing the risk of diabetes among this population. Therefore, this study points to the need for different targeted policies and programmes across various socioeconomic groups [44].

The government should launch a large-scale awareness programme to improve the early recognition of diabetes and reduce the stigma attached to it. There is a considerable need to understand the use of non-insulin therapies; school nurses should play a key role in educating children on their use.

### Strengths and limitations

Our study has certain strengths and limitations. To our knowledge, this study is the largest to date, consisting of a sample of nearly 0.2 million late adolescent respondents. It provides nationally representative estimates of hypertension and diabetes in India, stratified by sociodemographic and economic characteristics. Besides, it uses a validated cut-off for BP and differentiates between stage 1 and stage 2 hypertension care cascade among late adolescents [30–32].

Our study also has a few limitations that should be considered when interpreting the results. First, the cross-sectional nature of the NFHS-5 data limited our ability to establish causality or to observe temporal changes in hypertension and diabetes care cascades among late adolescents. This prevented us from assessing trends over time or making causal inferences about factors influencing prevalence or progression through the care cascade. Second, our reliance on single-day measurements for BP and blood glucose may not have accurately reflected long-term hypertension or diabetes status. Even though BP was measured three times at 5-minute intervals, temporary elevations due to factors like recent food intake, stress, or ‘white coat syndrome’ may have led to the misclassification of some participants as hypertensive. Similarly, random blood glucose testing may not have captured chronic hyperglycaemia as accurately as HbA1c testing can. Since our study was observational and not clinical, we could not measure the extent to which these factors may have influenced our estimates. Our approach was consistent with standard practices in community-based/observational studies and large-scale surveys like the Demographic and Health Surveys and NFHS. The Demographic and Health Surveys are globally rich data sources, and the biomarkers, such as BP measurement carried out in these surveys, are reliable [45]. We can conjecture that this limitation may have led to an overestimated prevalence for both conditions. Third, our ATC data was based on self-reporting, which is subject to recall bias. Participants may have inaccurately recalled their diagnosis or treatment history, potentially leading to under- or over-estimation of awareness and treatment levels. However, this is unlikely to happen in this age group [46]. Our classification of ‘treated’ group was based on participants reporting that they had sought treatment rather than confirming actual receipt or adherence to treatment. This may have led us to overestimate the proportion of adolescents receiving effective treatment for hypertension or diabetes. Fourth, we could not include early adolescents (10–14 years) in our analysis as the NFHS-5 did not collect data on hypertension and diabetes for this age group. This limited the comprehensiveness of our findings across the full adolescent age range. Fifth, while random blood glucose measurement was robust, the lack of HbA1c data, considered the gold standard for diagnosing diabetes, may have affected the accuracy of our diabetes prevalence estimates [21] and led to misclassification of some cases.

## CONCLUSIONS

In India, nearly one in four late adolescents have hypertension, while almost three in a hundred late have diabetes, with one in a hundred experiencing both. Around 90% are unaware, untreated, and uncontrolled. Given adolescents’ role in India's demographic dividend, urgent, tailored interventions are needed.

We recommend targeted screening in schools and colleges, integrated into health education, especially in high-prevalence regions. Awareness campaigns should address age-specific risk factors, and programmes must reach underprivileged communities, particularly in rural areas. Integrating NCD education, including hypertension and diabetes, into school curricula under the New Education Policy 2020 and National Curriculum Framework for School Education 2023 [35,40] is crucial. Schools should promote healthy lifestyles through better nutrition, physical activity, and stress management.

Future research should track adolescent health over time and assess policy effectiveness. By prioritising these measures, we can reduce the burden of hypertension and diabetes and protect India’s future workforce.

## Additional material


Online Supplementary Document
