# Supplementary material for: Care cascades of diabetes and hypertension among late adolescents in India
Source: J Glob Health. 2025 Mar 7;15:04101. doi: 10.7189/jogh.15.04101 (PMC11884645; doi:10.7189/jogh.15.04101)
Supplement: Online Supplementary Document [file jogh-15-04101-s001.pdf]

Supplement to: Malik BK, Goyal AK, Maiti S, Mohanty SK. Care cascades of diabetes and hypertension among late adolescents in India. J Glob Health. 2025;15:04101.

Supplementary Material

Table of Contents

Figure S1: Concentration curves of hypertension, diabetes, and comorbid hypertension and /or diabetes among late adolescents in India, 2019-21 1

Figure S2: Concentration curves of awareness, treatment and control of diabetes among late adolescents in India, 2019-21 2

Figure S3: Concentration curves of awareness, treatment and control of hypertension among late adolescents in India, 2019-21 2

Figure S4: Average values of diastolic blood pressure, systolic blood pressure and blood glucose among late adolescents by age, 2019-21 3

Figure S5: Comparison of diabetes prevalence, awareness, treatment and control among late adolescents and 20+ population in India. 2019-21 4

Figure S6: Comparison of hypertension prevalence, awareness, treatment and control among late adolescents and 20+ population in India. 2019-21 4

Table S1: Blood Pressure cut-offs across various ages 5

Table S2: Sample characteristics of late adolescents in India, 2019-21 (N=204,346) 6

Table S3: Mean systolic blood pressure, diastolic blood pressure and blood glucose level among the late adolescents in India, 2019-21 7

Table S4: Age-sex adjusted prevalence of diabetes and/or hypertension among late adolescents in India, 2019-21 8

Table S5: Prevalence, awareness, treatment and control of Stage 1 and Stage 2 hypertension among the late adolescents in India, 2019-21 9

Table S6: Mean of systolic blood pressure, diastolic blood pressure and blood glucose level for self-reports and measured among the late adolescents in India, 2019-21 9

Table S7: State-wise estimates of age-sex-adjusted prevalence of diabetes and hypertension among late adolescents in India, 2019-21 10

Table *S8*: Average marginal effects on the probability of diabetes among late adolescents in India, 2019-21 11

Table *S9*: Average marginal effects on the probability of hypertension among late adolescents in India, 2019-21 12

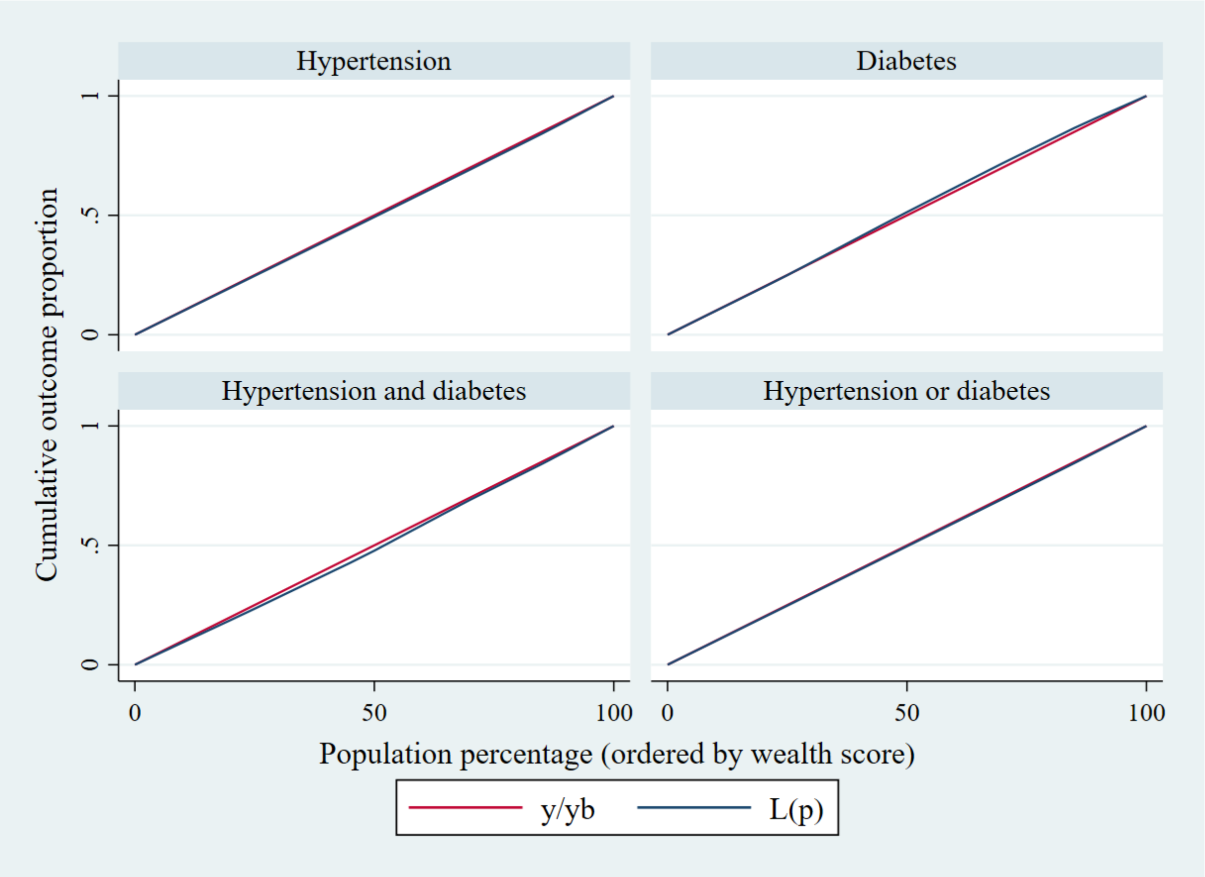

Figure S1: Concentration curves of hypertension, diabetes, and comorbid hypertension and /or diabetes among late adolescents in India, 2019-21

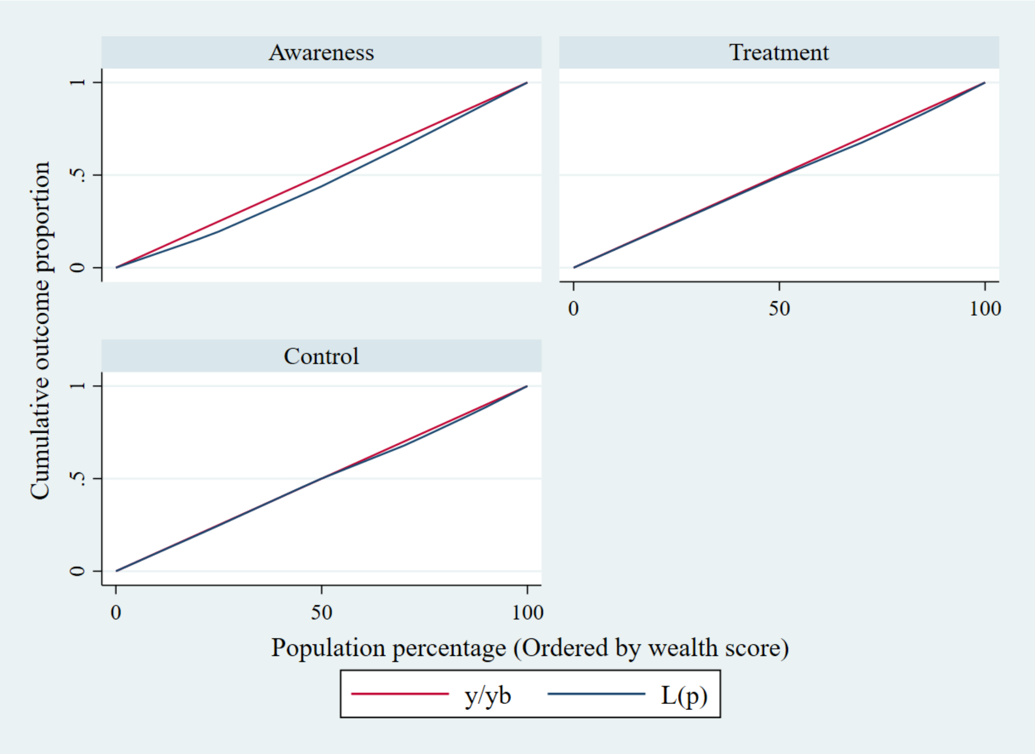

Figure S2: Concentration curves of awareness, treatment and control of diabetes among late adolescents in India, 2019-21

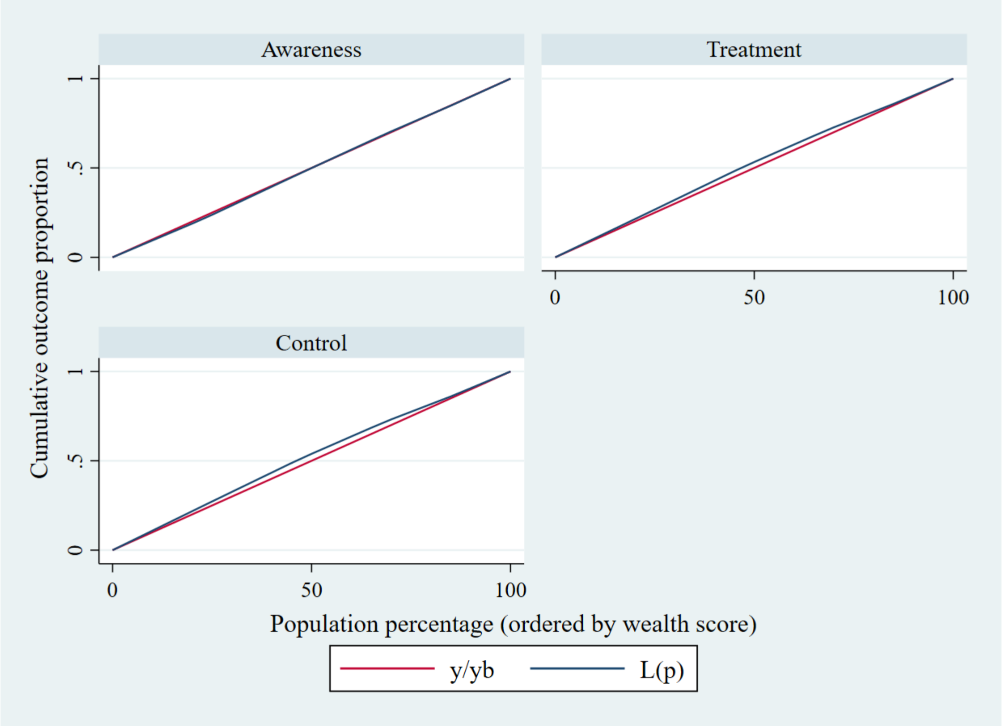

Figure S3: Concentration curves of awareness, treatment and control of hypertension among late adolescents in India, 2019-21

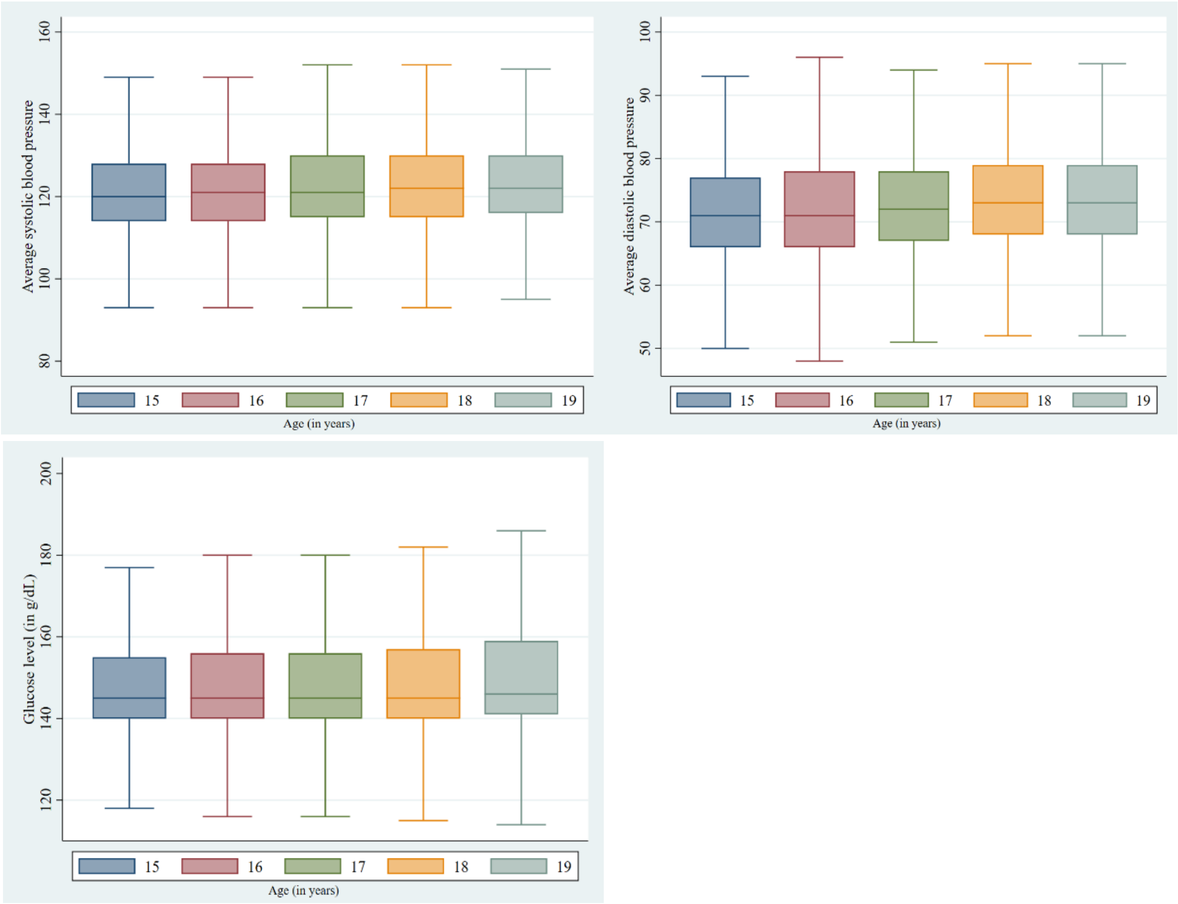

Figure S4: Average values of diastolic blood pressure, systolic blood pressure and blood glucose among late adolescents by age, 2019-21

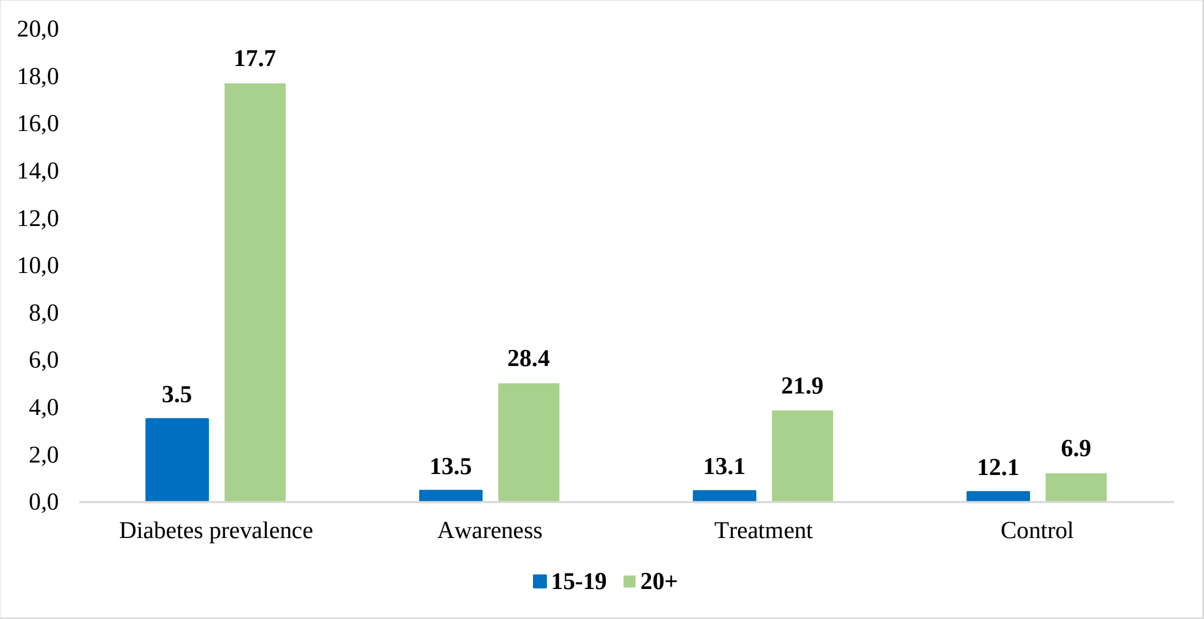

Figure S5: Comparison of diabetes prevalence, awareness, treatment and control among late adolescents and 20+ population in India. 2019-21

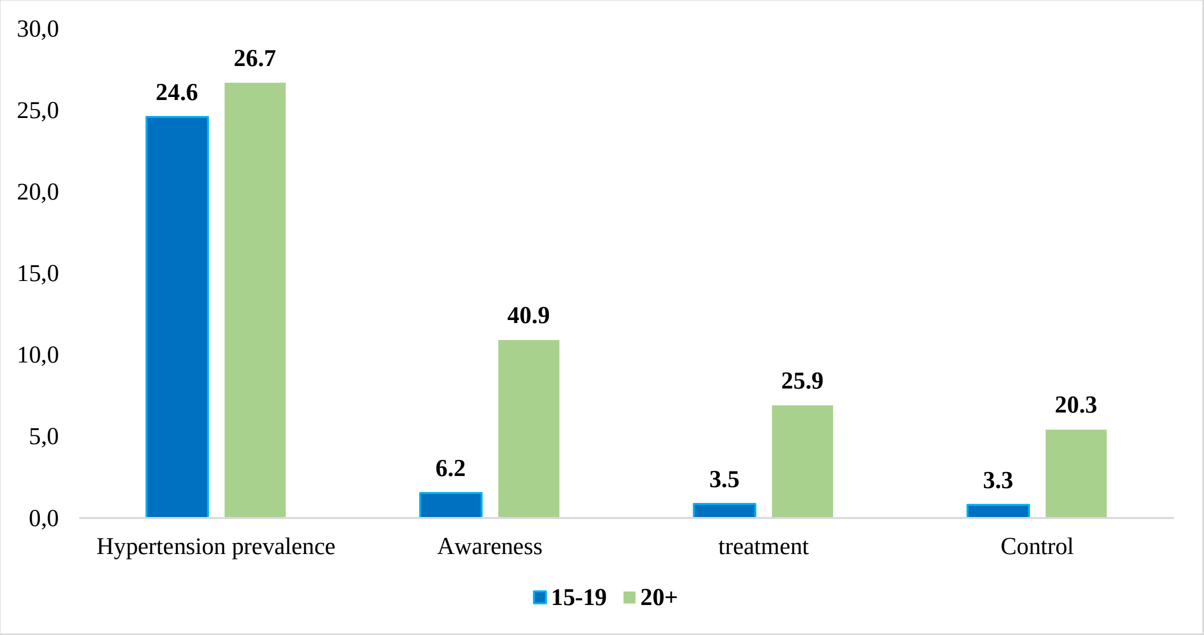

Figure S6: Comparison of hypertension prevalence, awareness, treatment and control among late adolescents and 20+ population in India. 2019-21

Table S1: Blood Pressure cut-offs across various ages

	For children 13 years to 19 years	For adults 18+
**Normal BP**	Systolic BP < 120 and diastolic BP < 80 mmHg	Systolic BP < 120 and diastolic BP < 80 mmHg
**Elevated BP**	Systolic BP 120 to 129 and diastolic BP < 80 mmHg	Systolic BP 120 to 139 and diastolic BP < 89 mm of Hg
**Stage 1 hypertension**	130/80 mmHg to 139/89 mmHg	140/90 mm of Hg to 159/99 of Hg
**Stage 2 hypertension**	≥ 140/90 mmHg	≥ 160/110 mm of Hg
**Source**	i) Flynn et al. (2017)<br>ii) Pediatrics (2022)	

Table S2: Sample characteristics of late adolescents in India, 2019-21 (N=204,346)

Socio-demographic characteristics	N	%[a]
**Age**		
15	41977	20.4
16	40846	19.8
17	39485	19.4
18	46150	22.7
19	35888	17.8
**Household Size**		
<3	24086	11.9
4-6	128777	62.5
7+	51483	25.5
**Sex**		
Female	108166	53.2
Male	96180	46.8
**Residence**		
Urban	45571	28.4
Rural	158775	71.6
**Education Level**		
No Education	7236	3.6
Primary	11234	5.4
Secondary	173910	84.2
Higher Secondary and above	11966	6.8
**Caste**		
Schedule Caste (SC)	44919	24.5
Schedule Tribe (ST)	40559	10.5
OBC	81814	45.0
Others	37054	20.0
**Religion**		
Hindu	158984	83.0
Muslim	21943	12.4
Christan	14353	2.2
Others	9066	2.4
**Currently Married**		
No	190343	92.4
Yes	14003	7.6
**Wealth quintile**		
Poorest	47839	21.5
Poorer	49746	22.6
Middle	43698	21.4
Richer	35657	19.1
Richest	27406	15.5
**Tobacco Use**		
No	195152	96.4
Yes	9194	3.6
**Alcohol Consumption**		
No	201189	98.9
Yes	3157	1.1
**Health Insurance**		
No	113074	58.0
Yes	91272	42.0

Note: Percentages are weighted, sample size is unweighted

Table S3: Mean systolic blood pressure, diastolic blood pressure and blood glucose level among the late adolescents in India, 2019-21

Socio-Demographic Characteristics	Systolic Blood Pressure mm of Hg x̄ (SE)	Diastolic Blood Pressure mm of Hg x̄ (SE)	Blood Glucose Level mg per dL x̄ (SE)
**Overall**	**136.7 (0.1)**	**82.0 (0.0)**	**158.5 (0.6)**
**Age**			
15	136.5 (0.3)	83.9 (0.1)	156.5 (1.3)
16	136.8 (0.3)	83.9 (0.1)	159.6 (1.4)
17	137.1 (0.3)	84.0 (0.1)	156.7 (1.1)
18	136.7 (0.2)	84.0 (0.1)	158.4 (1.4)
19	136.6 (0.2)	84.2 (0.1)	161.0 (1.7)
**Household Size**			
<3	136.4 (0.3)	84.1 (0.1)	157.6 (1.9)
4-6	136.8 (0.1)	84.0 (0.0)	158.7 (0.8)
7+	136.6 (0.3)	84.0 (0.1)	158.6 (1.2)
**Sex**			
Female	137.3 (0.3)	84.0 (0.0)	159.6 (0.9)
Male	136.5 (0.1)	84.0 (0.0)	157.4 (0.9)
**Residence**			
Urban	136.5 (0.2)	84.0 (0.1)	158.2 (1.1)
Rural	136.8 (0.1)	84.0 (0.0)	158.7 (0.8)
**Education Level**			
No Education	136.6 (0.6)	84.0 (0.2)	157.8 (2.0)
Primary	136.6 (0.5)	84.0 (0.1)	158.1 (2.2)
Secondar	136.7 (0.1)	84.0 (0.0)	158.5 (0.7)
Higher Secondary and above	137.1 (0.4)	84.3 (0.1)	159.6 (2.6)
**Caste**			
Schedule Caste	136.8 (0.3)	84.0 (0.1)	158.8 (1.4)
Schedule Tribe	136.5 (0.3)	84.0 (0.1)	157.1 (1.5)
OBC	136.9 (0.2)	84.1 (0.0)	158.3 (0.9)
Others	136.5 (0.2)	83.9 (0.1)	159.4 (1.5)
**Religion**			
Hindu	136.8 (0.1)	84.0 (0.0)	157.9 (0.7)
Muslim	136.4 (0.3)	84.0 (0.1)	160.5 (1.6)
Christan	136.5 (0.7)	83.8 (0.2)	165.0 (7.7)
Others	137.6 (0.6)	84.0 (0.1)	164.9 (4.8)
**Currently Married**			
No	136.7 (0.1)	84.0 (0.0)	158.5 (0.6)
Yes	136.8 (0.4)	84.0 (0.1)	159.4 (3.0)
**Wealth quintile**			
Poorest	136.8 (0.3)	84.0 (0.1)	156.9 (0.9)
Poorer	137.1 (0.3)	84.0 (0.1)	157.5 (1.1)
Middle	136.4 (0.2)	84.1 (0.1)	158.2 (1.2)
Richer	136.6 (0.3)	84.0 (0.1)	160.8 (2.1)
Richest	136.8 (0.3)	84.0 (0.1)	160.8 (1.9)
**Tobacco Use**			
No	136.8 (0.1)	84.0 (0.0)	158.6 (0.7)
Yes	136.1 (0.4)	84.1 (0.2)	157.1 (2.7)
**Alcohol Use**			
No	136.8 (0.1)	84.0 (0.0)	158.6 (0.6)
Yes	135.5 (0.5)	84.0 (0.2)	152.5 (1.9)
**Health Insurance**			
No	136.7 (0.2)	84.0 (0.0)	158.1 (0.8)
Yes	136.8 (0.2)	84.0 (0.1)	159.2 (1.0)

Table S4: Age-sex adjusted prevalence of diabetes and/or hypertension among late adolescents in India, 2019-21

Socio-Demographic Characteristics	Prevalence (N=204,346)			
	Diabetes and Hypertension (%)	(95% CI)	Diabetes or Hypertension (%)	(95% CI)
**Overall**	1.06	0.99, 1.12	27.87	27.60, 28.15
**Age**				
15	0.72	0.62, 0.83	23.24	22.73, 23.75
16	0.91	0.78, 1.03	25.48	24.96, 26.00
17	0.87	0.74, 0.99	27.3	26.77, 27.87
18	1.3	1.15, 1.46	30.6	30.08, 31.11
19	1.51	1.33, 1.68	33	32.41, 33.62
**Household Size**				
<3	1.24	1.04, 1.45	28.68	27.96, 29.39
4-6	1.00	0.93, 1.07	27.57	27.26, 27.89
7+	1.18	1.02, 1.34	28.71	28.06, 29.36
**Sex**				
Female	0.91	0.83, 0.99	24.8	24.44, 25.11
Male	1.22	1.12, 1.32	31.3	30.95, 31.73
**Residence**				
Urban	1.13	0.98, 1.27	28.12	27.51, 28.74
Rural	1.03	0.96, 1.10	27.78	27.47, 28.09
**Education Level**				
No Education	0.86	0.57, 1.15	30.24	28.93, 31.55
Primary	0.96	0.75, 1.16	29.47	28.42, 30.52
Secondary	1.07	1.00, 1.14	27.74	27.44, 28.04
Higher Secondary and above	1.05	0.81, 1.29	27.12	26.15, 28.10
**Caste**				
Schedule Caste	1.03	0.90, 1.15	27.44	26.91, 27.97
Schedule Tribe	1.07	0.90, 1.24	30.26	29.58, 30.93
OBC	1.08	0.99, 1.18	26.78	26.37, 27.19
Others	1.02	0.89, 1.16	28.52	27.91, 29.14
**Religion**				
Hindu	1.06	0.98, 1.13	27.24	26.94, 27.55
Muslim	1.04	0.86, 1.22	28.9	28.04, 29.77
Christian	1.21	0.80, 1.61	29.91	28.73, 31.08
Others	1.08	0.78, 1.39	33.62	32.27, 34.96
**Currently Married**				
No	1.03	0.97, 1.10	27.91	27.62, 28.20
Yes	1.34	1.08, 1.60	27.44	26.52, 28.36
**Wealth quintile**				
Poorest	1.03	0.90, 1.15	27.73	27.21, 28.25
Poorer	0.95	0.83, 1.07	27.73	27.23, 28.24
Middle	1.13	0.99, 1.27	27.69	27.14, 28.23
Richer	1.08	0.93, 1.23	27.74	27.14, 28.34
Richest	1.12	0.94, 1.30	28.68	27.95, 29.40
**Tobacco Use**				
No	1.06	0.99, 1.13	27.86	27.58, 28.15
Yes	1.00	0.74, 1.26	28.17	27.03, 29.31
**Alcohol Use**				
No	1.06	0.99, 1.12	27.83	27.54, 28.11
Yes	0.88	0.49, 1.27	30.97	29.03, 32.91
**Health Insurance**				
No	1.03	0.95, 1.12	28.61	28.24, 28.97
Yes	1.09	0.99, 1.18	26.9	26.56, 27.34

Table S5: Prevalence, awareness, treatment and control of Stage 1 and Stage 2 hypertension among the late adolescents in India, 2019-21

Hypertension	Stage 1 (%, 95% CI)	Stage 2 (%, 95% CI)
Prevalence	24.3% (24.0, 24.6)	5.2 (5.1 - 5.4)
Awareness	6.2 (5.8, 6.5)	27.1 (25.8 - 28.4)
Treatment	3.5 (3.3, 3.7)	15.8 (14.8 - 16.8)
Control	3.3 (3.1, 3.5)	15.5 (14.6 - 16.5)

Note: CI: confidence interval

Table S6: Mean of systolic blood pressure, diastolic blood pressure and blood glucose level for self-reports and measured among the late adolescents in India, 2019-21

Average	Only Measured (95% CI)	Only Self-reported (95% CI)
Systolic Blood pressure	122.8 (122.7, 123.0)	112.2 (111.6, 112.8)
Diastolic Blood Pressure	82.8 (82.8, 82.9)	73.2 (72.7, 73.6)
Glucose Level	160.1 (158.8, 161.4)	114.6 (110.1, 119.1)

Note: CI: confidence interval

Table S7: State-wise estimates of age-sex-adjusted prevalence of diabetes and hypertension among late adolescents in India, 2019-21

States	Diabetes % (95% CI)	Hypertension % (95% CI)	Diabetes or Hypertension % (95% CI)	Diabetes and Hypertension % (95% CI)
Jammu & Kashmir	1.8 (1.3, 2.2)	27.9 (25.4, 30.3)	28.9 (26.4, 31.4)	0.7 (0.4, 1.0)
Himachal Pradesh	2.8 (1.9, 3.7)	24.6 (21.7, 27.5)	26.6 (23.8, 29.5)	0.7 (0.3, 1.2)
Punjab	2.4 (1.9, 2.9)	29.2 (27.6, 30.8)	30.8 (29.1, 32.4)	0.8 (0.5, 1.1)
Chandigarh	1.0 (0.0, 2.5)	24.3 (16.1, 32.5)	24.9 (16.8, 32.9)	0.4 (0.0, 1.3)
Uttarakhand	2.3 (1.7, 2.8)	33.6 (31.6, 35.7)	34.9 (32.8, 36.9)	1.0 (0.6, 1.4)
Haryana	3.7 (3.1, 4.2)	29.6 (28.2, 31.1)	32.1 (30.6, 33.5)	1.2 (0.9, 1.5)
Delhi	1.8 (1.0, 2.5)	32.7 (30.6, 34.7)	33.7 (31.6, 35.7)	0.8 (0.4, 1.1)
Rajasthan	2.1 (1.8, 2.4)	27.8 (26.7, 28.8)	29.1 (28.0, 30.2)	0.7 (0.6, 0.9)
Uttar Pradesh	3.1 (2.9, 3.4)	29.4 (28.7, 30.1)	31.4 (30.8, 32.1)	1.1 (1.0, 1.2)
Bihar	4.4 (4.1, 4.8)	19.0 (18.2, 19.9)	22.4 (21.5, 23.3)	1.1 (0.9, 1.3)
Sikkim	4.7 (1.7, 7.6)	36.8 (30.2, 43.4)	38.3 (31.7, 44.8)	3.2 (0.5, 5.9)
Arunachal Pradesh	3.4 (2.7, 4.1)	36.1 (34.1, 38.1)	37.5 (35.5, 39.6)	1.9 (1.4, 2.5)
Nagaland	2.2 (1.3, 3.0)	26.7 (24.0, 29.5)	28.2 (25.4, 30.9)	0.8 (0.3, 1.2)
Manipur	3.9 (2.9, 4.8)	29.1 (26.2, 32.0)	31.8 (28.9, 34.7)	1.2 (0.7, 1.8)
Mizoram	3.1 (1.6, 4.5)	32.2 (28.3, 36.1)	34.2 (30.2, 38.2)	1.1 (0.4, 1.8)
Tripura	5.2 (3.9, 6.4)	23.4 (20.7, 26.2)	27.0 (24.1, 29.9)	1.6 (0.9, 2.3)
Meghalaya	3.6 (2.8, 4.3)	29.1 (27.0, 31.2)	31.3 (29.2, 33.4)	1.4 (0.9, 1.9)
Assam	4.3 (3.6, 5.0)	20.4 (19.0, 21.8)	23.5 (22.1, 25.0)	1.2 (0.9, 1.5)
West Bengal	5.9 (5.1, 6.7)	18.1 (16.6, 19.6)	22.7 (21.0, 24.5)	1.3 (0.9, 1.7)
Jharkhand	3.5 (3.0, 4.1)	27.6 (26.3, 28.9)	29.5 (28.1, 30.8)	1.7 (1.2, 2.1)
Odisha	3.7 (3.1, 4.2)	23.7 (22.4, 25.0)	26.4 (25.2, 27.7)	0.9 (0.7, 1.2)
Chhattisgarh	2.6 (2.2, 3.0)	30.5 (28.9, 32.0)	32.1 (30.5, 33.6)	1.0 (0.8, 1.3)
Madhya Pradesh	3.1 (2.8, 3.4)	25.4 (24.4, 26.3)	27.6 (26.6, 28.6)	0.9 (0.7, 1.1)
Gujarat	4.7 (4.0, 5.3)	18.9 (17.7, 20.0)	22.6 (21.4, 23.7)	0.9 (0.7, 1.2)
Dadra and Nagar Haveli	5.2 (2.8, 7.6)	21.4 (16.8, 25.9)	25.6 (20.7, 30.5)	0.9 (0.0, 1.9)
Maharashtra	3.0 (2.6, 3.5)	24.7 (23.3, 26.0)	26.8 (25.5, 28.2)	0.9 (0.6, 1.1)
Andhra Pradesh	4.1 (3.2, 4.9)	16.6 (14.9, 18.3)	19.8 (18.1, 21.5)	0.9 (0.5, 1.3)
Karnataka	3.4 (2.8, 4.0)	22.1 (20.5, 23.6)	24.0 (22.5, 25.6)	1.4 (0.9, 1.9)
Goa	4.4 (1.4, 7.4)	22.8 (16.1, 29.6)	26.0 (18.8, 33.1)	1.3 (0.0, 3.3)
Lakshadweep	3.2 (1.4, 5.0)	16.0 (11.2, 20.8)	18.4 (13.6, 23.2)	0.8 (0.0, 2.0)
Kerala	2.9 (2.2, 3.5)	17.6 (15.9, 19.4)	19.8 (18.1, 21.6)	0.7 (0.4, 1.0)
Tamil Nadu	4.0 (3.4, 4.6)	22.9 (21.5, 24.3)	25.1 (23.6, 26.6)	1.8 (1.4, 2.2)
Puducherry	4.0 (1.8, 6.1)	21.0 (16.4, 25.5)	24.5 (19.7, 29.2)	0.5 (0.0, 1.1)
Andaman and Nicobar	2.2 (0.4, 3.9)	24.3 (17.5, 31.0)	25.2 (18.2, 32.2)	1.2 (0.0, 2.5)
Telangana	3.4 (2.8, 4.0)	20.8 (19.4, 22.2)	23.3 (21.9, 24.6)	0.9 (0.6, 1.3)
Ladakh	4.4 (1.6, 7.2)	41.6 (33.9, 49.2)	42.7 (35.4, 50.1)	3.2 (0.6, 5.8)

Note: CI: confidence interval

Table *S8*: Average marginal effects on the probability of diabetes among late adolescents in India, 2019-21

SES Characteristics	Diabetes Prevalence	Awareness	Treatment	Control
	AME (95% CI)	AME (95% CI)	AME (95% CI)	AME (95% CI)
**Age**	**0.003*** (0.002, 0.004)**	-0.005 (-0.013, 0.003)	-0.005 (-0.015, 0.004)	-0.006(-0.016,0.003)
**Sex**				
Female	ref	ref	ref	ref
Male	**0.003** (0.000, 0.005)**	-0.002 (-0.024, 0.020)	0.003 (-0.021, 0.027)	0.005(-0.018,0.028)
**Education level**				
No Education	ref	ref	ref	ref
Primary	-0.001 (-0.008, 0.006)	**0.079** (0.019, 0.020)**	0.028 (-0.025, 0.081)	0.029(-0.023,0.080)
Secondary	0.003 (-0.003, 0.009)	**0.051** (0.010, 0.092)**	**0.054** (0.012, 0.095)**	**0.047** (0.006,0.087)**
Higher Secondary and above	0.004 (-0.004, 0.011)	**0.063** (0.005, 0.121)**	0.019 (-0.034, 0.072)	0.018(-0.035,0.070)
**Marital Status**				
Unmarried	ref	ref	ref	ref
Married	0.003 (-0.002, 0.007)	0.043 (-0.003, 0.090)	-0.035(-0.072,0.002)	-0.027(-0.065,0.010)
**Place of residence**				
Urban	ref	ref	ref	ref
Rural	0.001 (-0.003, 0.004)	-0.003 (-0.035, 0.028)	-0.004(-0.041,0.033)	-0.010(-0.047,0.027)
**Religion**				
Hindu	ref	ref	ref	ref
Muslim	0.001 (-0.003, 0.006)	-0.016 (-0.051, 0.019)	-0.003(-0.042,0.035)	-0.010(-0.048,0.027)
Christian	-0.003 (-0.010, 0.003)	0.087 (-0.016, 0.190)	0.038(-0.046,0.123)	0.017(-0.060,0.095)
Others	-0.002 (-0.008, 0.004)	0.022(-0.049,0.093)	0.032(-0.038,0.102)	0.006(-0.058,0.071)
**Caste**				
Schedule Caste	ref	ref	ref	ref
Schedule Tribe	-0.001 (-0.005, 0.003)	0.000 (-0.045,0.046)	-0.006(-0.043,0.031)	0.001(-0.035,0.038)
Other Backward Class	0.002 (-0.001, 0.005)	-0.001(-0.030,0.028)	0.005(-0.024,0.034)	0.001(-0.027,0.029)
Others	**0.005** (0.001, 0.009)**	**-0.036** (-0.071,-0.001)**	0.007(-0.030,0.044)	0.009(-0.028,0.046)
**Household Size**				
1-3	ref	ref	ref	ref
4-6	-0.002 (-0.006, 0.002)	-0.027(-0.063,0.009)	-0.008(-0.044,0.028)	-0.007(-0.042,0.028)
7+	0.003 (-0.002, 0.007)	-0.031(-0.074,0.012)	0.003(-0.047,0.053)	-0.001(-0.050,0.049)
**Wealth quintile**				
Poorest	ref	ref	ref	ref
Poorer	0.000 (-0.004, 0.003)	**0.031** (0.005,0.058)**	0.001(-0.033,0.034)	0.009(-0.024,0.042)
Middle	-0.004 (-0.008, 0.000)	**0.060*** (0.028,0.092)**	-0.010(-0.047,0.028)	-0.011(-0.048,0.026)
Richer	**-0.006** (-0.010, -0.002)**	**0.075*** (0.039,0.111)**	-0.011(-0.053,0.031)	-0.012(-0.052,0.029)
Richest	**-0.008** (-0.013, -0.004)**	**0.078** (0.033,0.122)**	0.011(-0.040,0.062)	0.011(-0.039,0.061)
**Tobacco Use**				
No	ref	ref	ref	ref
Yes	-0.001 (-0.007, 0.004)	0.051(-0.019,0.121)	0.068(-0.003,0.140)	0.057(-0.012,0.125)
**Alcohol Use**				
No	ref	ref	ref	ref
Yes	-0.006 (-0.016, 0.003)	-0.047(-0.128,0.033)	**-0.077** (-0.141, -0.013)**	**-0.068** (-0.132, -0.003)**
**Health Insurance**				
No	ref	ref	ref	ref
Yes	0.000 (-0.003, 0.002)	0.020(-0.004,0.043)	0.004(-0.020,0.028)	0.003(-0.021,0.026)

Note: CI-Confidence Interval; Marginal effects in bold are significant; AME: Average Marginal Effect
**** p < 0.001, ** p < 0.05, * p < 0.10*

Table *S9*: Average marginal effects on the probability of hypertension among late adolescents in India, 2019-21

SES Characteristics	Hypertension Prevalence	Awareness	Treatment	Control
	AME (95% CI)	AME (95% CI)	AME (95% CI)	AME (95% CI)
**Age**	**0.025*** (0.023,0.027)**	0.000(-0.002,0.002)	**-0.003(-0.005,-0.001)**	**-0.003(-0.004,-0.001)**
**Sex**				
Female	ref	ref	ref	ref
Male	**0.062*** (0.057,0.067)**	**-0.033*** (-0.039,-0.027)**	**-0.016*** (-0.021,-0.011)**	**-0.017*** (-0.022,-0.013)**
**Education level**				
No Education	ref	ref	ref	ref
Primary	0.000(-0.017,0.016)	0.011(-0.003,0.026)	0.003(-0.009,0.015)	0.002(-0.009,0.014)
Secondary	**-0.014* (-0.028,0.000)**	**0.018** (0.007,0.029)**	0.007(-0.002,0.016)	0.006(-0.003,0.015)
Higher Secondary and above	**-0.019** (-0.036,-0.001)**	**0.023** (0.007,0.039)**	0.004(-0.009,0.017)	0.002(-0.011,0.014)
**Marital Status**				
Unmarried	ref	ref	ref	ref
Married	**-0.011(-0.021,-0.001)**	**0.077*** (0.061,0.093)**	**0.018** (0.007,0.029)**	**0.017** (0.007,0.028)**
**Place of residence**				
Urban	ref	ref	ref	ref
Rural	-0.006(-0.014,0.002)	-0.002(-0.012,0.008)	0.003(-0.004,0.009)	0.001(-0.006,0.007)
**Religion**				
Hindu	ref	ref	ref	ref
Muslim	0.008(-0.002,0.018)	-0.007(-0.017,0.002)	-0.002(-0.009,0.005)	-0.001(-0.008,0.006)
Christian	**-0.025** (-0.041, -0.009)**	0.010(-0.014,0.034)	**0.027** (0.005,0.050)**	**0.028** (0.006,0.051)**
Others	**0.044*** (0.028,0.060)**	**0.024** (0.005,0.043)**	0.001(-0.014,0.016)	0.001(-0.014,0.016)
**Caste**				
Schedule Caste	ref	ref	ref	ref
Schedule Tribe	**0.027*** (0.017,0.037)**	-0.010(-0.021,0.000)	-0.001(-0.008,0.007)	-0.002(-0.009,0.006)
Other Backward Class	-0.007(-0.014,0.000)	**0.008* (0.000,0.016)**	**0.007** (0.001,0.012)**	**0.006** (0.001,0.012)**
Others	0.000(-0.009,0.009)	-0.004(-0.013,0.006)	0.005(-0.003,0.012)	0.004(-0.003,0.011)
**Household Size**				
1-3	ref	ref	ref	ref
4-6	-0.008(-0.016,0.000)	0.000(-0.008,0.009)	0.002(-0.005,0.010)	0.001(-0.006,0.009)
7+	-0.002(-0.012,0.008)	0.002(-0.009,0.012)	-0.004(-0.013,0.005)	-0.006(-0.015,0.003)
**Wealth quintile**				
Poorest	ref	ref	ref	ref
Poorer	0.003(-0.004,0.010)	0.005(-0.004,0.014)	-0.005(-0.012,0.002)	-0.005(-0.012,0.002)
Middle	**0.010** (0.002,0.018)**	0.004(-0.005,0.013)	**-0.009** (-0.016, -0.001)**	**-0.009** (-0.017, -0.002)**
Richer	**0.018*** (0.008,0.027)**	-0.007(-0.017,0.004)	**-0.011** (-0.019, -0.002)**	**-0.012** (-0.020, -0.003)**
Richest	**0.030*** (0.019,0.041)**	0.003(-0.009,0.016)	**-0.010** (-0.019,0.000)**	**-0.011** (-0.020, -0.001)**
**Tobacco Use**				
No	ref	ref	ref	ref
Yes	-0.003(-0.016,0.010)	0.001(-0.016,0.017)	-0.007(-0.019,0.005)	-0.007(-0.019,0.005)
**Alcohol Use**				
No	ref	ref	ref	ref
Yes	**0.026** (0.003,0.050)**	0.000(-0.032,0.032)	**-0.015** (-0.028, -0.002)**	**-0.015** (-0.027, -0.003)**
**Health Insurance**				
No	ref	ref	ref	ref
Yes	**-0.009** (-0.015, -0.004)**	-0.007(-0.013,0.000)	-0.001(-0.005,0.004)	-0.001(-0.006,0.003)

Note: CI- confidence interval; Marginal effects in bold are significant; AME: Average Marginal Effect
**** $p < 0.001$, ** $p < 0.05$, * $p < 0.10$*

References

1. Flynn JT, Kaelber DC, Baker-Smith CM, Blowey D, Carroll AE, Daniels SR, et al. Clinical Practice Guideline for screening and Management of high blood pressure in children and adolescents. Pediatrics. 2017;140(3). Available from: https://doi.org/10.1542/peds.2017-1904

2. Indian Academy of Pediatrics (IAP), Kumar R, Saxena V, Gupta P, Kinjawadekar U, Soans S, et al. STANDARD TREATMENT GUIDELINES 2022: Hypertension. Indian Academy of Pediatrics. 2022. https://iapindia.org/pdf/Ch-057-stg-hypertension.pdf
